# K^+^ regulates Ca^2+^ to drive inflammasome signaling: dynamic visualization of ion flux in live cells

**DOI:** 10.1038/cddis.2015.277

**Published:** 2015-10-29

**Authors:** J R Yaron, S Gangaraju, M Y Rao, X Kong, L Zhang, F Su, Y Tian, H L Glenn, D R Meldrum

**Affiliations:** 1 Center for Biosignatures Discovery Automation, The Biodesign Institute, Arizona State University, Tempe, 85287 AZ, USA; 2 Biological Design Graduate Program, School of Biological and Health Systems Engineering, Ira A. Fulton Schools of Engineering, Arizona State University, Tempe, 85287 AZ, USA

## Abstract

P2X_7_ purinergic receptor engagement with extracellular ATP induces transmembrane potassium and calcium flux resulting in assembly of the NLRP3 inflammasome in LPS-primed macrophages. The role of potassium and calcium in inflammasome regulation is not well understood, largely due to limitations in existing methods for interrogating potassium in real time. The use of KS6, a novel sensor for selective and sensitive dynamic visualization of intracellular potassium flux in live cells, multiplexed with the intracellular calcium sensor Fluo-4, revealed a coordinated relationship between potassium and calcium. Interestingly, the mitochondrial potassium pool was mobilized in a P2X_7_ signaling, and ATP dose-dependent manner, suggesting a role for mitochondrial sensing of cytosolic ion perturbation. Through treatment with extracellular potassium we found that potassium efflux was necessary to permit sustained calcium entry, but not transient calcium flux from intracellular stores. Further, intracellular calcium chelation with BAPTA-AM indicated that P2X_7_-induced potassium depletion was independent of calcium mobilization. This evidence suggests that both potassium efflux and calcium influx are necessary for mitochondrial reactive oxygen generation upstream of NLRP3 inflammasome assembly and pyroptotic cell death. We propose a model wherein potassium efflux is necessary for calcium influx, resulting in mitochondrial reactive oxygen generation to trigger the NLRP3 inflammasome.

NLR family, pyrin domain-containing 3 (NLRP3) is the most extensively studied among the inflammasome family of caspase-1-activating complexes and is critical to the innate immune response to infection, damage and pathophysiological dysfunction.^1^ A likely reason for the widespread interest in NLRP3 is its responsiveness to extracellular ATP, pore-forming toxins, biological particulate matter, synthetic nanoparticles, vaccine adjuvants and pathogens including bacteria, fungi and viruses.^2, 3, 4, 5, 6, 7^ There is ongoing debate about how such a diverse array of stimuli are able to converge on a single pathway, despite the apparent differences in their mode of action.

Proposed mechanisms for regulating the activation of the NLRP3 inflammasome pathway are varied and controversial.^1^ Among the most popular proposed mechanisms is the flux of cellular ions. The asymmetric distribution of ions in cellular compartments establishes a gradient such that, under conditions of membrane permeability, ions rapidly diffuse across the gradient without energy input.^8^ Cells benefit from asymmetric ion distribution by using it to affect rapid processes such as neuronal action potentials.^8^ Recent work has implicated potassium flux as the common trigger in regulating NLRP3 inflammasome activity.^9^ Indeed, it has been understood for over two decades that potassium flux regulates the processing of interleukin (IL)-1β, a downstream effect of inflammasome activation.^10, 11^ While potassium is the most commonly studied ion posited to regulate the NLRP3 pathway, calcium flux has gained popularity in recent years because intervention in calcium mobilization has inhibitory effects on inflammasome activity.^12, 13, 14^ Both ions are permeant to the non-specific cation channel formed by plasma membrane expressed purinergic receptor P2X, ligand-gated ion channel, 7 (P2X_7_) which is activated by external ATP. However, it is currently not known how or whether the two ions relate to each other in the context of inflammasome regulation.^1, 12^

In addition to ion flux, mitochondrial reactive oxygen species (mROS) signaling has been proposed as a critical regulator of NLRP3 activation.^15^ Mitochondrial dysfunction and loss of mitochondrial membrane potential lead to a rapid increase in mROS production, which has been described to activate the inflammasome through the activity of thioredoxin-interacting protein (TXNIP).^16^ In support of this mechanism, most known NLRP3-activating stimuli induce ROS generation and specific mitochondria-targeted ROS scavengers have been shown to inhibit inflammasome assembly.^17^ The existence of a convergent pathway involving ion flux, particularly of potassium, and ROS generation in triggering the assembly of the inflammasome has been suggested, however such a link has remained elusive.^18, 19^

In this study, we tested the hypothesis that P2X_7_ purinergic receptor activation with extracellular ATP induces mitochondrial ROS generation and this effect is mediated by cytosolic and mitochondrial potassium depletion. We applied a novel intracellular potassium sensor to characterize the real-time dynamics of potassium mobilization in the mouse macrophage cell line J774A.1 after stimulation with ATP. By co-localizing the sensor signal to mitochondria using a mitochondria-specific dye, we observed a P2X_7_-dependent mitochondrial potassium depletion that was sensitive to pharmacological and ionic inhibition. Temporally, mitochondrial potassium mobilization occurred before potassium efflux-dependent mitochondrial ROS generation. Further study identified a critical role for calcium influx upstream of mitochondrial ROS generation, inflammasome assembly and pro-inflammatory cytokine release. We report here the first-ever multiplexed imaging of intracellular potassium and calcium in live cells and our finding that potassium efflux is required for sustained calcium influx, while calcium chelation had no effect on the kinetics of potassium efflux. We propose that mitochondrial ROS generation is a downstream effect of potassium efflux-dependent calcium influx and defines a coordinated, ion flux-driven regulation of the NLRP3 inflammasome via oxidative signaling.

## Results

### P2X_7_ receptor-dependent potassium efflux induces inflammasome activation in J774A.1 macrophages

**Figure 1:**
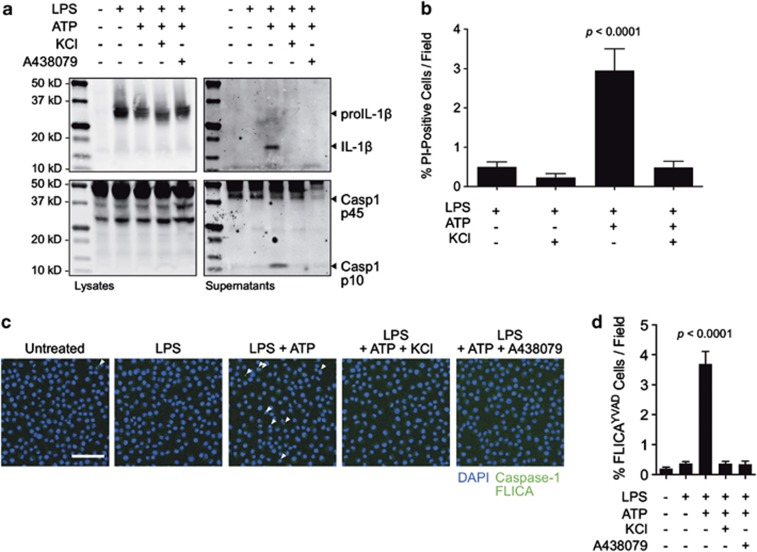
P2X_7_-induced potassium efflux regulates NLRP3 inflammasome assembly and pyroptotic cell death. (**a**) Immunoblot analysis of procaspase-1 p45 and activated p10 fragments, and proIL-1β (34 kD) and mature (17 kD) fragments in the lysates and concentrated supernatants of J774A.1 cells primed for 4 h with 1 *μ*g/ml LPS and stimulated with 3 mM ATP for 30 min with or without addition of 130 mM extracellular KCl or 25 *μ*M of the P2X_7_ antagonist A438079. (**b**) Propidium iodide in J774A.1 cells primed with LPS for 4 h and stimulated with ATP in the presence or absence of 130 mM extracellular KCl. (**c**) Caspase-1 FLICA staining (green) in J774A.1 cells untreated or primed for 4 h with 1 *μ*g/ml LPS and subsequently stimulated with 3 mM ATP for 30 min with or without 130 mM extracellular KCl or 25 *μ*M A438079. Arrows: caspase-1 specks indicative of inflammasome assembly. Scale bar represents 50 *μ*M. Nuclei are stained with NucBlue Fixed DAPI solution (blue). (**d**) Image cytometry analysis of inflammasomes detected by Caspase-1 FLICA. J774A.1 cells untreated or primed for 4 h with 1 *μ*g/ml LPS and subsequently stimulated with 3 mM ATP for 30 min with or without 130 mM extracellular KCl or 25 *μ*M A438079. Bar graph represents mean counts and standard error from at least 4000 cells for each condition from at a minimum of three fields in two independent experiments. Statistics were calculated by one-way ANOVA with Tukey's *post hoc* analysis

Our first objective was to determine the response of the J774A.1 mouse monocyte/macrophage cell line to extracellular ATP. As expected, immunoblotting indicated that untreated J774A.1 lacks proIL-1β while maintaining constitutive levels of procaspase-1 (Figure 1a). Upon priming with *E. coli* lipopolysaccharide (LPS), proIL-1β protein becomes highly expressed. Release of active caspase-1 p10 and mature IL-1β p17 was detected in concentrated supernatants of LPS-primed J774A.1 after treatment with 3 mM extracellular ATP. The release of both active components was abolished in the presence of high extracellular potassium (to suppress the intracellular–extracellular concentration gradient) as well as the selective, competitive, P2X_7_ receptor antagonist A438079.^20^ The requirement for potassium efflux in inflammasome-mediated pyroptotic cell death was confirmed by propidium iodide staining and live-cell imaging (Figure 1b). Combined LPS and ATP treatment resulted in a time-dependent accumulation of cells positive for propidium iodide that was inhibited in the presence of 130 mM extracellular potassium. Further, tagging of activated caspase-1 with the fluorescent marker FLICA revealed the assembly of the inflammasome as indicated by the presence of classical perinuclear caspase-1-positive specks that were significantly suppressed by high extracellular potassium and treatment with A438079 (Figures 1c and d). We performed co-localization experiments to confirm that the triggered structure was the NLRP3 inflammasome and contained ASC as expected (Supplementary Figure S1). Thus, J774A.1 exhibits the 1st/2nd signal (LPS priming and ATP stimulation, respectively) behavior representative of the potassium efflux-dependent NLRP3 inflammasome pathway in macrophages.

### ATP-induced calcium influx regulates the NLRP3 inflammasome

**Figure 2:**
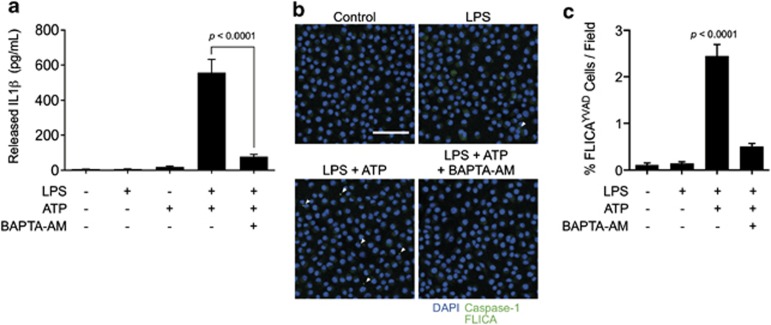
Calcium influx is an upstream regulator of IL-1β release and NLRP3 inflammasome assembly. (**a**) ELISA analysis of released IL-1β from J774A.1 cells primed with 1 *μ*g/ml LPS for 4 h and stimulated with 3 mM ATP for 30 min. Where indicated, cells were pretreated with 100 *μ*M BAPTA-AM before addition of ATP. Statistics were calculated by one-way ANOVA with Tukey's *post hoc* and represent the mean and standard error of two independent experiments. (**b**) Cells were prepared as in (**a**), except for the addition of caspase-1 FLICA 1 h before the addition of ATP (green). Arrows indicate perinuclear caspase-1 specks. Blue fluorescence indicates nuclei stained with NucBlue Fixed DAPI solution. Scale bar represents 50 *μ*M. (**c**) Image cytometry analysis of inflammasomes detected by caspase-1 FLICA. Bar graph represents mean counts and standard error from at least 4000 cells for each condition from at a minimum of three fields in two independent experiments. Statistics were calculated by one-way ANOVA with Tukey's *post hoc* analysis

We next sought to determine the role of ATP-induced calcium influx on inflammasome activation. A previous study has shown that intracellular calcium chelation with BAPTA-AM suppresses IL-1β processing and release upon ATP-induced inflammasome activation.^14^ In agreement with this observation, we found that BAPTA-AM significantly suppressed ATP-induced IL-1β processing and release as indicated by ELISA in J774A.1 cell supernatants (Figure 2a). It has not yet been reported whether calcium chelation suppresses IL-1β processing and release upstream or downstream of inflammasome assembly, though some reports propose a possible calcium influx-dependent lysosomal exocytosis pathway for IL-1β release.^21^ FLICA was used to observe ATP-induced inflammasome assembly as indicated by perinuclear caspase-1 specks. While stimulation with 3 mM ATP resulted in perinuclear caspase-1 speck appearance indicative of inflammasome assembly, chelation with BAPTA-AM significantly inhibited inflammasome formation (Figures 2b and c). These results suggest that calcium influx regulates ATP-induced NLRP3 activation upstream of inflammasome formation.

### Direct visualization of potassium mobilization in macrophages with a novel intracellular sensor

**Figure 3:**
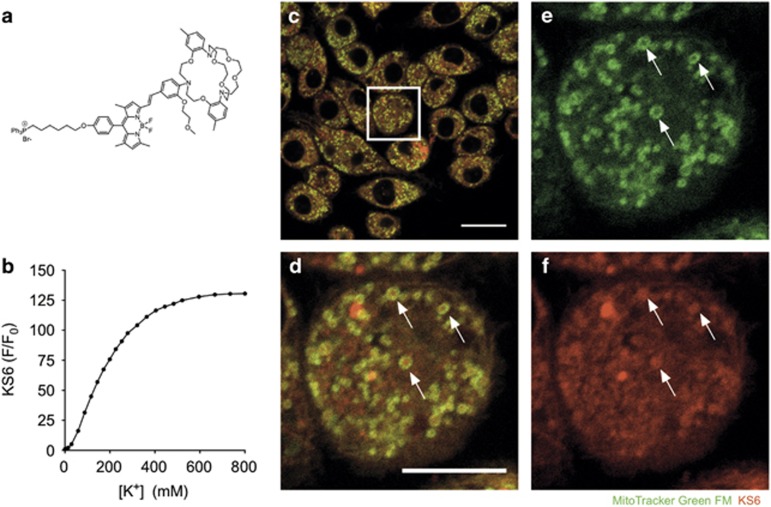
KS6 localizes to predominantly mitochondria in live cells. (**a**) Chemical structure of the intracellular potassium sensor KS6. (**b**) Spectrofluorophotometric characterization of KS6 sensor signal response to potassium titration in solution. (**c**) J774A.1 cells were stained with KS6 intracellular potassium sensor and MitoTracker Green FM before imaging by confocal microscopy. (**d**) Inset of boxed region from (**c**) displaying the overlap of MitoTracker Green FM and KS6. (**e**) Signal from MitoTracker Green FM. (**f**) Signal from KS6. Arrows indicate discrete mitochondria clearly stained for both probes. Scale bar represents 20 *μ*M in (**c**) and 10 *μ*M in (**d**)

To better understand the intracellular potassium dynamics triggered by ATP-induced NLRP3 inflammasome activation, we utilized KS6 (Figure 3a), a novel intracellular potassium sensor that predominantly localizes to mitochondria, although background staining can be variably observed in other cellular compartments in a concentration- and cell type-specific manner.^22^ KS6 is sensitive to potassium in the physiological range (Figure 3b). Further, KS6 is highly selective for potassium against other biological monovalent and divalent ions such as iron(II), iron(III), copper, manganese, calcium, zinc, magnesium and sodium.^22^ We first confirmed the sensor localization in J774A.1 cells. Live-cell imaging revealed a strongly enriched signal from KS6 in the mitochondrial matrix as verified by co-staining with MitoTracker Green FM. Co-localization analysis with the CoLoc2 FIJI plugin determined a Spearman's rank correlation value of 0.799, indicating a predominantly mitochondrial localization (Figures 3c–f). Initial studies characterizing the performance of the sensor in HeLa and U87 cells at concentrations of 2 *μ*M yielded predominant co-localization with mitochondria with only minor background staining in other cellular compartments.^22^ Based on consultation with the sensor development team, 5 *μ*M was chosen as the appropriate concentration for J774A.1 cells. We found that loading with 5 *μ*M KS6 enabled dynamic potassium imaging of both mitochondrial and cytosolic potassium content with no phenotypic effect on cell viability in J774A.1 cells. Thus, KS6 is suitable for detection of subcellular potassium content.

**Figure 4:**
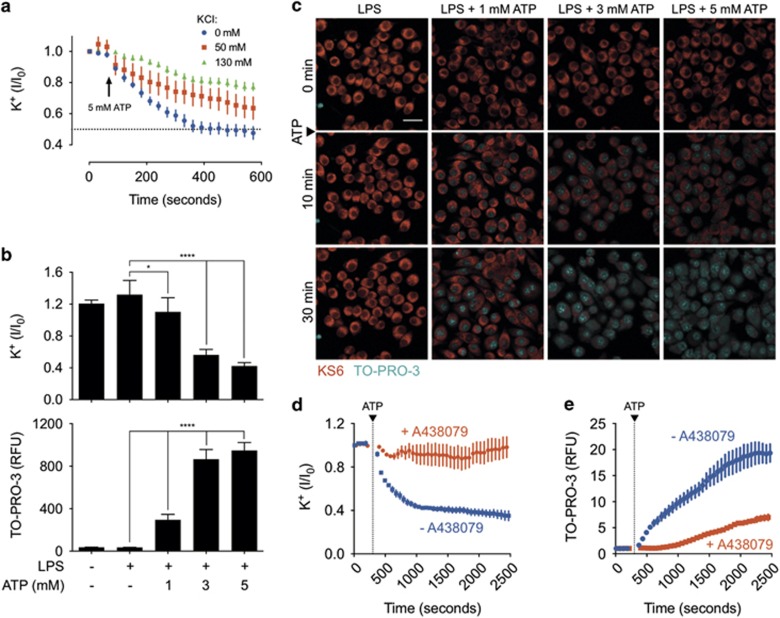
Real-time P2X_7_-dependent intracellular potassium dynamics observed with KS6. (**a**) Kinetic trace of potassium efflux from J774A.1 cells stimulated with 5 mM ATP at the indicated time point in the presence of 0 mM additional KCl (normal DMEM medium), 50 or 130 mM additional extracellular KCl. Traces represent the mean and standard error of 10–20 cells in each field. (**b**) Response at 40 min of potassium efflux (top panel) or TO-PRO-3 uptake (bottom panel) of J774A.1 cells primed for 4 h with 1 *μ*g/ml LPS and treated with 1, 3 or 5 mM extracellular ATP. Bars represent mean and standard deviation of 20 cells in each condition. Statistics were performed by one-way ANOVA with Fischer's LSD comparison test. *Indicates *P*<0.05 and **** indicates *P*<0.0001. (**c**) Representative fields at the indicated time points of LPS-primed J774A.1 loaded with KS6 (red) and treated with 1, 3 or 5 mM extracellular ATP in the presence of TO-PRO-3 (cyan). Scale bar represents 50 *μ*M. Pre-treatment of LPS-primed, ATP-stimulated J774A.1 macrophages with the P2X_7_ inhibitor A438079 suppresses (**d**) potassium efflux (**e**) and membrane permeability. Traces represent mean and standard error for five representative cells. Results are representative of at least two experiments

We next confirmed that whole-cell KS6 signal responds to ATP-induced P2X_7_ activation. P2X_7_ engagement results in the opening of a non-specific cation pore and potassium efflux down the intracellular–extracellular potassium concentration gradient.^23^ We probed this response by demonstrating a live-cell titration between normal (130 mM) and intermediate (50 mM) concentrations of extracellular potassium added to the cell culture medium (Figure 4a). In control experiments, we found that addition of equimolar (130 mM) concentrations of extracellular sodium had no effect on KS6 sensor response, indicating insensitivity to osmolarity effects (Supplementary Figure S2). Next, we tested whether differing concentrations of extracellular ATP would result in a dose-dependent opening of the P2X_7_ pore. It was recently reported that membrane permeability is dose dependently related to P2X_7_ receptor activation, but it is unknown whether potassium efflux is similarly dose dependent.^24^ We observed a dose dependence of both potassium efflux and membrane permeability as indicated by the response in the KS6 potassium sensor and uptake of the membrane impermeable DNA dye TO-PRO-3, respectively (Figures 4b and c). Both events were dependent on P2X_7_ activity as inhibition with A438079 suppressed both events (Figures 4d and e). Importantly, our single cell microscopic data confirm previous reports that the threshold for potassium concentration required for ATP-induced inflammasome activity is approximately 50-60% of basal levels, corresponding to a total cellular potassium concentration of about 60–80 mM. Taken together, these experiments demonstrate the ability to directly visualize P2X_7_-dependent intracellular potassium dynamics in live macrophages.

### Extracellular ATP mobilizes mitochondrial potassium downstream of P2X_7_ engagement

**Figure 5:**
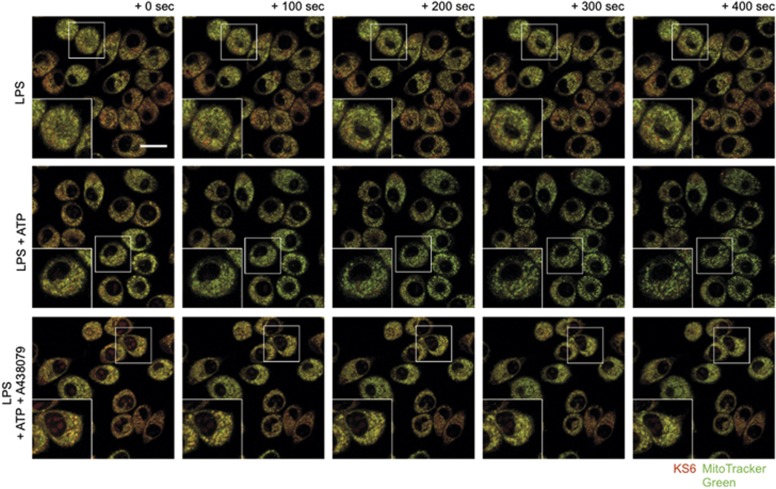
P2X_7_ activation results in mitochondrial potassium mobilization. J774A.1 cells were primed for 4 h with 1 *μ*g/ml LPS and loaded with 5 *μ*M KS6 (red) and 10 nM MitoTracker Green FM (green). Real-time confocal microscopy was performed to track the potassium dynamics after stimulation with 3 mM extracellular ATP with or without the P2X_7_ inhibitor A438079. Results revealed a rapid, receptor-dependent mobilization of potassium as indicated by a reduction in KS6 signal in the co-localized space with MitoTracker Green FM that was sensitive to inhibition with A438079. Subsequent to the mobilization, mitochondria appeared to fragment. Fields are representative of at least 3–5 experiments. Scale bar represents 20 *μ*M

Mitochondrial potassium represents a significant portion of total cellular potassium, as its concentration is nearly twice (200–300 mM) that of the cytosol (100–150 mM).^25^ Because extracellular ATP is detected at plasma membrane-localized P2X_7_ receptors resulting in cytosolic potassium efflux, we sought to determine whether the mitochondrial potassium pool was mobilized by macrophage purinergic signaling. We used KS6 and co-localized the signal with MitoTracker Green FM to observe the kinetics of mitochondrial potassium in live J774A.1 cells (Figure 5). Real-time monitoring of KS6 signal during P2X_7_ engagement with 3 mM extracellular ATP revealed both cytosolic and mitochondrial potassium depletion. The depletion of cytosolic and mitochondrial potassium was suppressed by 130 mM extracellular potassium or by inhibition of P2X_7_ with A438079. Notably, the loss of potassium occurred prior to the shrinking and disintegration morphology indicative of mitochondrial damage.

### Mitochondrial ROS generation is essential for pyroptosis in J774A.1 macrophages

**Figure 6:**
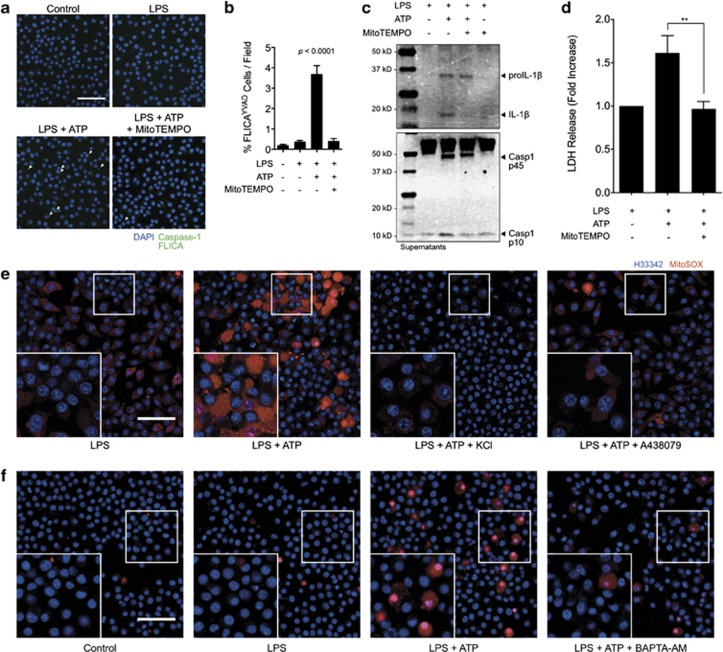
Mitochondrial ROS is essential for ATP-evoked inflammasome activity and is driven by P2X_7_-dependent cation flux in J774A.1 cells. (**a**) Caspase-1 FLICA staining (green) in J774A.1 cells primed for 4 h with 1 *μ*g/ml LPS and subsequently stimulated with 3 mM ATP for 30 min with or without 500 *μ*M MitoTEMPO treatment. Arrows point to caspase-1 specks indicative of inflammasome assembly. Scale bar represents 50 *μ*M. Nuclei are stained with NucBlue Fixed DAPI solution (blue). (**b**) Image cytometry analysis of inflammasomes detected by caspase-1 FLICA. Bar graph represents mean counts and standard error from at least 4000 cells for each condition from at a minimum of three fields in two independent experiments. Statistics were calculated by one-way ANOVA with Tukey's *post hoc* analysis. (**c**) Immunoblot analysis of pro-caspase-1 p45 and activated p10 fragments, and proIL-1β (34 kD) and mature (17 kD) fragments in the lysates and concentrated supernatants of J774A.1 cells primed for 4 h with 1 *μ*g/ml LPS and stimulated with 3 mM ATP for 30 min with or without pretreatment with 500 *μ*M MitoTEMPO. (**d**) Assessment of lactate dehydrogenase (LDH) activity in the supernatants of J774A.1 cells primed with 1 *μ*g/ml LPS and stimulated with ATP for 30 min with or without pretreatment with 500 *μ*M MitoTEMPO. Results are fold-change *versus* LPS primed cells and error bars represent standard error of two independent experiments. ** Indicates *P*<0.01 by one-way ANOVA with Tukey's *post hoc* comparison. (**e** and **f**) J774A.1 cells were left untreated, primed with LPS for 4 h, or primed with LPS and treated with 3 mM ATP as indicated. MitoSOX (red) was added to all samples 15 min after ATP addition and incubated for an additional 15 min before live-cell imaging. (**e**) Evaluation of the role of P2X_7_ and potassium efflux in mROS generation. 130 mM KCl and 25 *μ*M A438079 were added 15–20 min before ATP addition. (**f**) Evaluation of the role of calcium influx in mROS generation. 100 *μ*M BAPTA-AM was added 15–20 min before ATP addition. Nuclei are stained with Hoechst 33342 (blue). Scale bar is 50 *μ*m. Results are representative of at least two independent experiments

We next determined that mROS was necessary for the assembly and function of the inflammasome. LPS-primed J774A.1 cells were treated with ATP with and without pre-treatment with the mitochondria-localized reactive oxygen scavenger MitoTEMPO. Previous studies have shown that MitoTEMPO is effective in inhibiting pyroptosis and release of IL-1β.^17^ Here, we show that the assembly of the inflammasome speck, as indicated by FLICA labeling for caspase-1, is strongly inhibited in the presence of MitoTEMPO (Figures 6a and b). We further validate the need for mROS in inflammasome function by demonstrating an inhibition of caspase-1 p10 and IL-1β p17 processing and release when cells are treated with ATP in the presence of MitoTEMPO as detected by immunoblotting (Figure 6c). Finally, we demonstrate that pyroptotic cell death requires mROS by measurement of lactate dehydrogenase activity in cell supernatants (Figure 6d). These results demonstrate the need for mROS in the assembly and activity of the inflammasome.

### P2X_7_-dependent potassium and calcium ion flux is essential for mitochondrial mROS production

Direct visualization revealed that the mitochondrial potassium pool responds to receptor-mediated changes in intracellular potassium, hence, we sought to determine if ion flux had an effect on pro-inflammatory mitochondrial signaling. Using the mitochondria-targeted ROS probe MitoSOX, we investigated whether potassium efflux and calcium influx had an effect on ROS production (Figures 6e and f). Results indicated a substantial increase in MitoSOX signal when LPS-primed cells were stimulated with ATP. In support of a role for P2X_7_ signaling in this response, inhibition of the channel with A438079 reduced levels of mROS production to that of basal levels seen in LPS-primed J774A.1 cells. Importantly, both potassium efflux and calcium influx were necessary for the generation of mROS as treatment with 130 mM extracellular KCl (Figure 6e) or BAPTA-AM (Figure 6f) resulted in strong suppression of MitoSOX oxidation.

Comparing the kinetics of MitoSOX oxidation and potassium efflux in the mitochondria, we find that potassium mobilization is a rapid event and likely occurs upstream of ROS generation. While this is difficult to directly correlate due to KS6 exhibiting rapid response dynamics while MitoSOX converts by a comparatively slow process, the seconds-scale response of potassium efflux is notably quicker than the apparent minutes-scale generation of ROS. These results taken together suggest that calcium and potassium flux triggered by P2X_7_ activation results in mitochondrial ion imbalance and mROS generation upstream of NLRP3 inflammasome activation.

### ATP-induced potassium efflux is required for calcium influx

**Figure 7:**
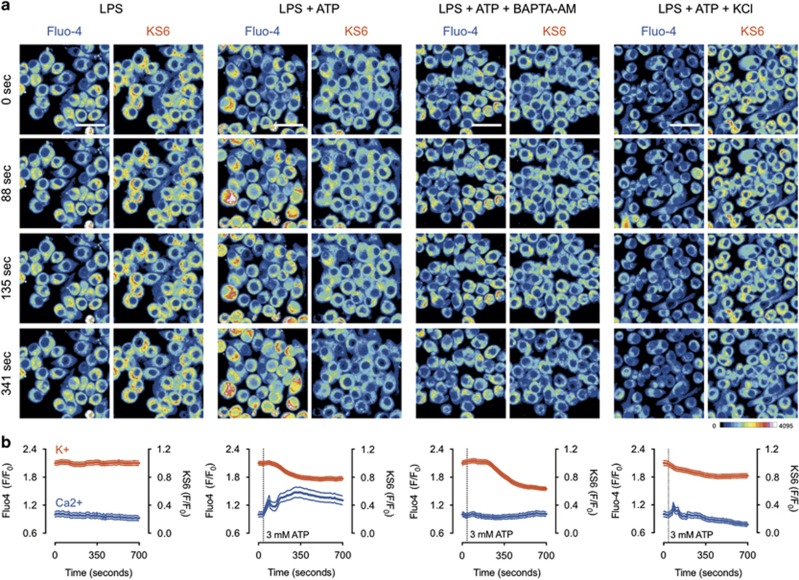
Real-time, multiplexed visualization of ATP-induced potassium and calcium dynamics. J774A.1 cells were primed for 4 h with 1 *μ*g/ml LPS, stained with KS6 and Fluo-4 DIRECT and both fluorophores were imaged simultaneously by confocal microscopy. (**a**) Representative fields for each condition showing Fluo-4 and KS6 signal responses. 16-color pseudocolor look-up tables were used for visualization. Blue indicates low signal intensity and red indicates high signal intensity. Scale bar represents 25 *μ*M. (**b**) Mean and standard error for 30 representative cells in each condition. Where indicated cells were stimulated with 3 mM ATP. Inhibitors were added 15–20 min before imaging. Results are representative of at least two experiments

While both potassium and calcium are implicated in ATP-induced inflammasome activation, it is unclear whether there is a relationship between them.^1^ To investigate the dynamics of ATP-induced ion flux, we performed multiplexed imaging of potassium and calcium by combining the KS6 intracellular potassium sensor with Fluo-4, a commercially available calcium indicator (Figure 7). Results showed that LPS priming alone had no dramatic effect on ion content; calcium transients were apparent but overall signal was stable for both calcium and potassium. Upon ATP stimulation, a rapid calcium signal spike occurred, followed by a second, more sustained increase in calcium signal. This bi-phasic calcium response is indicative of the kinetics associated with rapid, endoplasmic reticulum-stored calcium ahead of plasma membrane-localized calcium entry from the extracellular environment.^26^ Importantly, potassium flux occurred concurrently with the second phase of calcium influx, but was stable through the initial calcium spike. Calcium chelation with BAPTA-AM resulted in a suppression of calcium dynamics, but had no effect on the ability for ATP to induce potassium depletion. This suggests that potassium flux is upstream of calcium flux. Addition of extracellular potassium had no effect on the initial calcium spike after ATP addition, but suppressed the second, sustained calcium rise. Together, these results suggest that ATP-induced potassium efflux is upstream and necessary for plasma membrane-associated calcium influx, but not transient store-associated calcium spikes.

## Discussion

In this study, we investigated the question of how ion flux and mitochondrial reactive oxygen interact upstream of inflammasome assembly. Both of these phenomena are recognized as key mediators of inflammasome regulation and have been separately suggested as the common induction mechanism for inflammasome assembly. Despite the proposed link between potassium efflux and mitochondrial signaling resulting in inflammasome assembly, this association had yet to be observed.^1, 18, 19, 27^ Further, there has been limited evidence regarding the relationship between calcium and potassium. Thus, it has been unclear how ion flux and oxidative signaling contribute to inflammasome regulation.

The effect of potassium blockade is often used as a test for basic characterization of both new activators and inhibitors of the inflammasome because of its long-standing and apparently ubiquitous participation in linking stimulus detection and inflammasome assembly. Details of how potassium affects the inflammasome have not been adequately described, however, because of technical limitations in potassium measurement. The most common methods for determining the role of potassium in various aspects of the inflammasome pathway are either blockade with high extracellular potassium or quantitation of potassium content by spectroscopy or photometry of bulk populations lysed in nitric acid.^9, 18, 28^ While high extracellular potassium is effective for determining how blockade of potassium efflux affects downstream phenotypes, which we have also used in this study, it obscures intermediate responses and is unable to reveal cellular kinetics. Likewise, bulk cell potassium determination can provide only low-resolution kinetic details and completely obscures the contribution of individual cells or subpopulations in the response to stimuli. The latter point was recently identified to be an essential characteristic of inflammasome-associated response by macrophages. Specifically, it was observed by single cell analysis that IL-1β was released in a bursting manner only from cells dying by pyroptosis.^29^ This opposes the long-standing paradigm of secretion by various controversial pathways.^30^ This observation highlights the importance of investigating inflammasome-associated cellular processes at the single cell level and avoiding reliance on bulk cell determination methods.

While calcium indicators are well established and extensively used, existing methods for probing potassium are lacking. To date, Arlehamn *et al.*^31^ have reported the only live-cell imaging experiments on potassium in macrophages stimulated to undergo inflammasome assembly by infection with Pseudomonas. A major limitation to this study, however, is their application of PBFI for live cell potassium readout. PBFI is a potassium sensor that exhibits nearly equivalent sensitivity to sodium ions as it does to potassium ions, which makes its readout difficult to interpret.^32^ Further, PBFI has a Kd of <10 mM and its response saturates at approximately 50 mM, a concentration lower than the threshold for inflammasome activation as reported by others and reaffirmed in this study. Accordingly, upstream dynamics are obscured by a saturated sensor response and a depletion of cellular potassium can only be detected upon cell death with PBFI. Finally, PBFI is necessarily excited with UV light and therefore induces phototoxic damage to cells under observation, potentially obscuring the effects of stimulus-induced cell death. For the current studied we applied KS6, an improved intracellular potassium sensor, which addresses the drawbacks of PBFI.^22^ KS6 is excited by visible light, is strongly selective for potassium relative to sodium and other monovalent and divalent ions, readily taken up by live cells, and is sensitive to potassium across a supraphysiological range. Here we show that KS6 can be effectively used for observing dynamic responses of potassium in live cells and, further, that it can be multiplexed with other intracellular indicators for analytes such as calcium.

Detection of extracellular ATP by the P2X_7_ purinergic receptor is a prototypical stimulus for NLRP3 inflammasome activation.^10, 28^ Potassium efflux and ROS generation have independently been proposed as downstream effects of P2X_7_ activation.^33^ By live-cell imaging, we identified a rapid and robust mitochondrial potassium mobilization associated with P2X_7_ engagement. P2X_7_ is expressed on the plasma membrane and its sensing activity is therefore localized distally to mitochondrial responses.^34^ We propose that mitochondria mobilize their potassium pool as a response to changes in cytosolic potassium levels, which is directly responsive to P2X_7_ activity. This provides additional support for the observation that mitochondria respond to cytosolic potassium levels, as it was previously shown that mitochondria are capable of sequestering and buffering cytosolic potassium.^35^ A mitochondrial potassium buffering mechanism is further supported by our finding that efflux is ATP dose dependent. P2X_7_ has multiple ATP-sensing sites that have been shown to dose dependently affect the level of pore dilation permissive to ion flux.^36^ It is still not well understood how mitochondria respond to cytosolic potassium, but we speculate that this may occur either through mitochondrial potassium channels or transient leakage associated with membrane potential changes.^37^ A pharmacologic screen with readout from KS6 will help to identify channels and signaling pathways that result in mitochondrial sensitivity to cytosolic potassium. Our data suggest that the magnitude of P2X_7_ activation dictates the degree of potassium efflux, and therefore inflammasome assembly responses.

**Figure 8:**
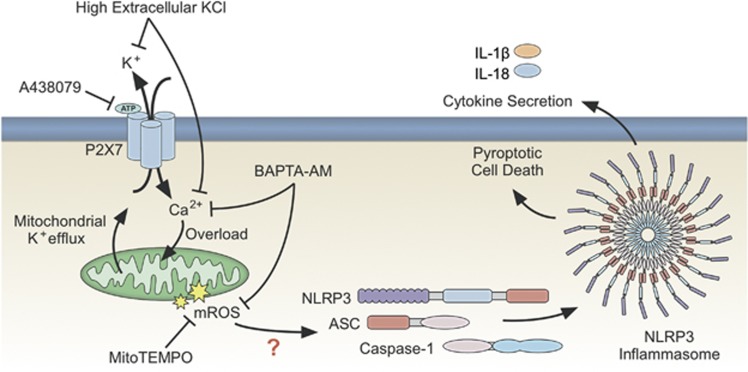
Proposed mechanism for ion flux-dependent regulation of the NLRP3 inflammasome. Activation of the P2X_7_ receptor pathway with extracellular ATP results in the exchange of potassium and calcium across the plasma membrane, dominantly regulated by efflux of potassium from the cytosol to the extracellular space. Influx of calcium causes a mitochondrial calcium overload resulting in mitochondrial destabilization and mROS generation, which activates the NLRP3 inflammasome through an unknown mechanism, but possibly by involvement of TXNIP or Syk.^6, 12, 16, 38, 39^ P2X_7_ receptor activation also results in a mitochondrial potassium efflux that may be involved in mitochondrial destabilization and mROS generation. Inhibition of potassium efflux prevents calcium influx and downstream mROS generation. Likewise, chelation of intracellular calcium prevents mROS generation

Intracellular ion homeostasis is essential for maintenance of mitochondrial integrity as mitochondrial membrane potential is heavily dependent on charge distribution.^8, 37^ We hypothesized that the mitochondrial potassium loss we observed would be correlated with mitochondrial reactive oxygen production. In support of this, we found that blockade with high extracellular potassium or the P2X_7_ inhibitor A438079 suppresses the elevated levels of mitochondrial ROS observed with ATP treatment. Likewise, we found that potassium blockade and calcium chelation suppress mROS generation. We report here the first-ever multiplexed imaging of potassium and calcium in live cells. This approach allowed us to demonstrate that potassium efflux is upstream of, and necessary for, sustained calcium influx in response to ATP treatment. These results suggest a mechanism whereby potassium efflux triggered by P2X_7_ activation regulates calcium influx, ultimately resulting in mROS generation leading to NLRP3 inflammasome activation, possibly through mitochondrial calcium overload (Figure 8). Additional studies may help to better define the potassium-sensitive link between mitochondrial signaling and NLRP3 inflammasome assembly. We propose that rapidly activating intermediates such as TXNIP or Syk may be appropriate targets for providing this link based on the association between both of these targets and mitochondrial ROS in the NLRP3 inflammasome signaling pathway.^6, 12, 16, 38, 39^

Recently, a study was published by Katsnelson *et al.*^40^ reporting that potassium efflux induced NLRP3 inflammasome assembly independently of calcium influx. Among the conclusions of this paper are that (1) potassium efflux is sufficient for inflammasome assembly independent of calcium flux and (2) calcium flux antagonists, such as BAPTA, inhibit the inflammasome by an off-target mechanism dissociated from their calcium-related effects. Here, we report that potassium efflux leads to calcium influx and that both ion fluxes are necessary for ATP-induced inflammasome assembly. To demonstrate this, we showed that inhibition of potassium efflux also inhibited calcium influx and that calcium chelation had no effect on potassium efflux. Our results do not directly contradict the results of Katsnelson *et al.* as they did not interrogate the effects of potassium efflux inhibition on calcium content and their calcium and potassium experiments were performed on independent populations of cells with disparate temporal resolution. Also, Katsnelson *et al.*^40^ fail to reproduce earlier data showing that inflammasome activity is inhibited in calcium-free medium as reported by Murakami *et al.*^13^ Regarding off-target inhibitor effects, Katsnelson *et al.* propose that chelation of zinc ions by BAPTA may cause calcium-independent inflammasome inhibition. This mechanism of inhibition would be contrary to the previously described promotion, not inhibition, of inflammasome assembly due to zinc ion chelation.^41^

This study establishes a previously unknown relationship between potassium and calcium during purinergic receptor-dependent activation of the NLRP3 inflammasome. Namely, potassium efflux is the dominant regulatory ion upstream of calcium influx, both of which are required for mitochondrial oxidative signaling leading to NLRP3 inflammasome activation. This finding reconciles the observation that intervention in calcium signaling can modulate inflammasome signaling, but treatment with calcium ionophores is insufficient for stimulating IL-1β processing and release.^10, 13^ This study also provides the first highly selective, real-time observation of ATP-induced potassium dynamics as well as the first multiplexed imaging of calcium and potassium in live cells. We propose that future work toward elucidating the NLRP3 inflammasome pathway will benefit from application of real-time visualization of potassium and calcium ion dynamics.

## Materials and Methods

### Cell culture

The mouse macrophage cell line J774A.1 (TIB-67) was obtained from ATCC (Manassas, VA, USA) and cultured in DMEM containing 10% FBS, 100 U/ml penicillin and 100 *μ*g/ml streptomycin (Gibco, Grand Island, NY, USA) at 37 °C with 5% CO_2_ in a humidified atmosphere. Cells were passaged by scraping and viability and density were assessed by Trypan Blue dye exclusion on a Countess automated cell counter (Life Technologies, Grand Island, NY, USA).

### KS6 potassium sensor loading

KS6 (ex/em 561/630 nm) was kept in a 1-mM DMSO stock solution stored at 4 °C. To facilitate consistent dye distribution, stock KS6 was combined 1:1 with 10% Pluronic F127 and mixed thoroughly by pipetting before loading.^42^ The mixture was added 1:100 to each well of a chamber slide for a final KS6 concentration of 5 *μ*M and incubated for 30–60 min at 37 °C. Where indicated, cells were subsequently stained with 10 nM MitoTracker Green FM (Molecular Probes, Eugene, OR, USA). Co-localization analysis was performed using ImageJ/FIJI version 1.49 f and the CoLoc 2 plugin.^43^ KS6 was developed in-house^22^ (Center for Biosignatures Discovery Automation, Arizona State University, Tempe, AZ, USA).

### Live-cell imaging

Cells seeded in an 8-chamber *μ*-slide (Ibidi, Verona, WI, USA) were primed with 1 *μ*g/ml *E. coli* O111:B4 LPS (Sigma-Aldrich, St. Louis, MO, USA) for 2–4 h followed by the addition of inhibitors or other treatment as indicated, then stimulation with 1–5 mM ATP (Sigma-Aldrich). Alternately, cells were stimulated by addition of 20 *μ*M nigericin (#11437, Cayman Chemical, Ann Arbor, MI, USA). Samples were imaged on a Nikon Ti microscope equipped with a C2si confocal scanner (Nikon Instruments, Melville, NY, USA) and Tokai Hit stage-top incubator (Tokai Hit Co., Shizuoka, Japan). Excitation laser lines were 408, 488, 561 and 639 nm and emission was collected by photomultipliers filtered for the standard DAPI, FITC, TRITC and Cy5 bandwidths. Objectives used were × 20 air 0.75 NA, × 60 oil immersion 1.4 NA or × 60 water immersion 1.2 NA, all from Nikon. Where indicated, cells were imaged in the presence of 5 *μ*M TO-PRO-3 (Molecular Probes). For calcium imaging, cells were loaded with 1 × Fluo-4 DIRECT solution (Molecular Probes) and incubated for 30–60 min before imaging. For analysis of mitochondrial ROS production, MitoSOX red (Molecular Probes) was added 15 min after stimulation with ATP.

### Caspase-1 FLICA assay

J774A.1 cells were seeded at a density of 1–2 × 10^5^ per well in 200 *μ*l of complete DMEM and grown overnight. The following day cells were primed for 4 h with 1 *μ*g/ml *E. coli* O111:B4 LPS. During the last hour of priming, cells were loaded with 1 × FAM-YVAD-FMK (Caspase-1 FLICA; Immunochemistry Technologies, Bloomington, MN, USA). Inhibitors, as indicated, were added during the last 15–20 min of priming. Cells were stimulated with 3 mM ATP for 30 min, subsequently washed 2 × with warm DMEM and fixed in 2% formaldehyde solution, prepared in PBS from powdered paraformaldehyde, for 10 min at room temperature. Cells were permeabilized in 0.25% Triton X-100 for 10 min, then counterstained with NucBlue DAPI fixed cell stain (Life Technologies), according to the manufacturer's instructions in PBS, then rinsed 2x with PBS and submerged in 200 *μ*l mounting medium (90% glycerol in PBS and 0.1% NaN_3_). Samples were imaged by laser-scanning confocal microscopy as a series of 0.5 *μ*M z-stacks on a Nikon Ti microscope equipped with a Nikon C2si confocal scanner controlled by the Nikon Elements AR software. Caspase-1 FLICA was excited at 488 nm and emission was collected in the FITC channel while NucBlue was excited at 408 nm and emission was collected in the DAPI channel. Stacks were prepared as maximum intensity projections using ImageJ/FIJI.

### Immunofluorescence

Cells seeded in an 8-chamber *μ*-slide were primed for 4 h with 1 *μ*g/ml *E. coli* O111:B4 LPS. Cells were additionally treated with the caspase-1 inhibitor ac-YVAD-CHO (50 *μ*M) for the last 30 min of priming to inhibit cell detachment downstream of inflammasome assembly. For inflammasome stimulation, cells were treated with 3 mM ATP for 1 h. Cells were fixed with 2% formaldehyde solution, permeabilized in 0.25% Triton X-100 in PBS and blocked in 0.25% Triton X-100 in PBS containing 5% BSA at room temperature. Polyclonal rabbit Caspase-1 p10 antibody (#SC-514, Santa Cruz Biotechnology, Dallas, TX, USA) was added 1 : 100 overnight at 4 °C. Secondary antibody, AlexaFluor 488-conjugated anti-rabbit secondary antibody (#A-11034, Life Technologies), was added 1 : 1000 at room temperature for 1 h. DAPI solution was added using NucBlue and samples were covered with 150 *μ*l mounting medium (90% glycerol, 10% (10 × ) PBS with 0.01% NaN_3_) and kept at 4 °C until imaging. Inflammasome images were obtained as 0.5–1 *μ*m z-stacks and presented as maximum intensity projections. For co-localization studies, after caspase-1 FLICA labeling (described above), cells were immunolabeled similarly except fixed with 4% formaldehyde and probed with NLRP3 antibody (#SC-66846, Santa Cruz Biotechnology) diluted 1 : 100 or ASC antibody (#AL-177, Adipogen, San Diego, CA, USA) diluted 1:200 overnight, followed by AlexaFluor 568-conjugated anti-rabbit secondary antibody (#A-11036, Life Technologies) diluted 1 : 1000.

### Image cytometry

Confocal z-stacks for nuclear and FLICA channels were opened in FIJI (ImageJ v1.49 f, http://www.fiji.sc) and converted to maximum intensity projections. Automatically detected Otsu thresholding was applied to each channel resulting in a positive signal mask. To each mask, a binary watershed algorithm was applied to separate closely neighboring objects. To get the final count for nuclei and FLICA, the Analyze Particles function was applied to each channel with a size threshold of 20 to infinity square pixels for nuclei and 5 to infinity square pixels for FLICA foci. The results were analyzed in GraphPad Prism by one-way ANOVA with Tukey's *post hoc* analysis.

### Lysate and supernatant protein collection

Cells were seeded in 6-well plates (10^6^ cells/well) and primed for 4 h with 1 *μ*g/ml *E. coli* O111:B4 LPS in complete DMEM containing 10% FBS. After priming, cells were washed 1 × with serum-free DMEM and 1.1 ml of warm serum-free DMEM was added to each well. Where noted, cells were treated with inhibitors for 15–30 min. Inflammasome activation was triggered by application of freshly prepared 3 mM ATP solution in serum-free DMEM for 30 min. After stimulation, supernatants were collected and spun at 14 000 ×  *g* for 15 min at 4 °C to remove cellular debris and approximately 1 ml was transferred to fresh 1.5 ml tubes. Ten microliters of StrataClean resin (Agilent, Santa Clara, CA, USA) was added to each supernatant, mixed well and placed on a rotator in a 4 °C refrigerator for 1 h. Concentrated supernatant protein was collected by pelleting the StrataClean resin, removing the supernatant and heating the resin resuspended in 50 *μ*l 1 × Laemmli buffer at 95 °C for 5 min. Cell lysates were prepared by addition of 100 *μ*l hot 1 × Laemmli buffer to each well for 5–10 min, scraping and transferring samples to 1.5 ml tubes and heating at 95 °C for 5 min.

### Immunoblotting

Twelve microliters of concentrated supernatant or lysate was separated on 4–12% Mini-Protean TGX gels (Bio-Rad, Hercules, CA, USA) at 100 V for 1 h. Proteins were transferred onto 0.2 *μ*m nitrocellulose membranes (LiCor, Lincoln, NE, USA) at 100 V for 1 h, and subsequently blocked in 5% non-fat dry milk in PBS containing 0.2% Tween-20 for 1 h. Blocked membranes were incubated in 5% BSA in PBS containing 0.2% Tween-20 and either 1 : 500 rabbit polyclonal against Caspase-1 p10 (#SC-514, Santa Cruz) or 1 : 1000 goat polyclonal against IL-1β (#AF-401-NA, R&D Systems, Minneapolis, MN, USA) and rotated overnight at 4 °C. The following day, donkey anti-goat IRDye 800CW and goat anti-rabbit IRDye 680RD secondary antibodies (Li-Cor) were applied at a dilution of 1 : 15 000 with rocking for 1 h at room temperature. Membranes were imaged on a Li-Cor Odyssey CLx on auto exposure with high quality setting.

### Lactate dehydrogenase release assay

Cells were seeded in 96-well plates and primed for 4 h with 1 *μ*g/ml *E. coli* O111:B4 LPS. Cells were treated for the last 15–30 min with 500 *μ*M MitoTEMPO and stimulated for 30 min with 3 mM ATP. Fifty microliters of supernatant was used for LDH activity assay with the CytoTox96 Non-Radioactive Cytotoxicity Kit (Promega, Madison, WI, USA) according to the manufacturer's instructions.

### ELISA

J774A.1 cells were seeded in 96-well plates at a concentration of 10^5^ cells/well and incubated overnight. Cells were primed for 4 h with 1 *μ*g/ml *E. coli* O111:B4 LPS and subsequently stimulated for 30 min with 3 mM ATP in 100 *μ*l medium. Where indicated, cells were treated with 100 *μ*M BAPTA-AM (Tocris, Minneapolis, MN, USA) for 15 min before ATP treatment. Supernatants were collected and released IL-1β was evaluated with ELISA using the R&D Systems DuoSet kit according to the manufacturer's protocol. Developed plates were read on a Biotek Synergy H4 mutli-mode plate reader with Gen5 software.

### Statistical analysis

Statistics were performed where indicated with GraphPad Prism version 6 (GraphPad, La Jolla, CA, USA) and procedures for each analysis are described in the figure captions.

## Abbreviations

NLRP3: NLR family, pyrin domain-containing 3
IL-1β: interleukin-1 beta
P2X_7_: purinergic receptor P2X, ligand-gated ion channel, 7
ROS: reactive oxygen species
mROS: mitochondrial reactive oxygen species
TXNIP: thioredoxin-interacting protein
LPS: lipopolysaccharide
