# Supplementary figures and images for: K+ regulates Ca2+ to drive inflammasome signaling: dynamic visualization of ion flux in live cells
Source: Cell Death Dis. 2015 Oct 29;6(10):e1954–. doi: 10.1038/cddis.2015.277 (PMC5399176; doi:10.1038/cddis.2015.277)

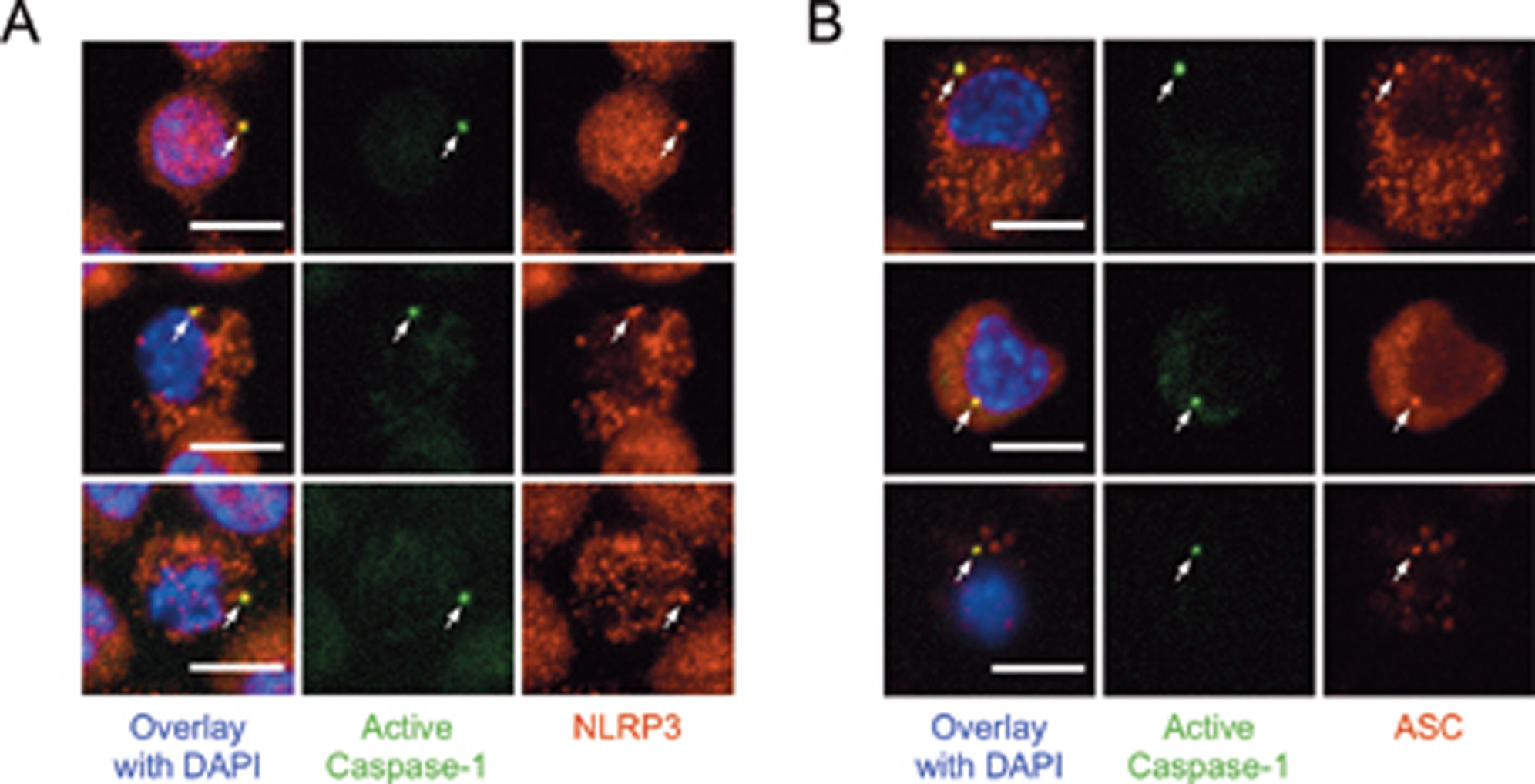

Supplement: Supplementary Figure S1 [file cddis2015277x1.tif]

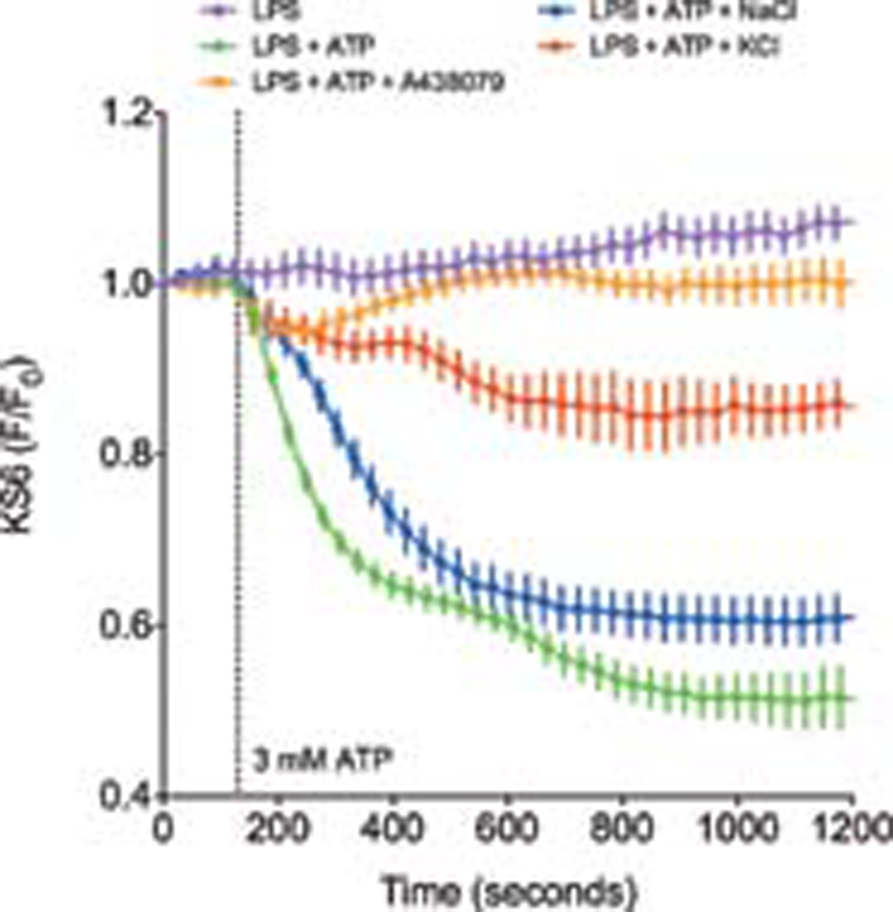

Supplement: Supplementary Figure S2 [file cddis2015277x2.tif]
