# Supplementary material for: K+ regulates Ca2+ to drive inflammasome signaling: dynamic visualization of ion flux in live cells
Source: Cell Death Dis. 2015 Oct 29;6(10):e1954–. doi: 10.1038/cddis.2015.277 (PMC5399176; doi:10.1038/cddis.2015.277)
Supplement: Supplementary Figure Legends [file cddis2015277x3.docx]

**Figure S1. LPS and ATP stimulate NLRP3 inflammasome assembly in J774A.1 macrophages.** LPS-primed J774A.1 macrophage cells were primed for 4 hours with 1 µg/mL E. coli O111:B4 LPS prior to stimulation with 3 mM extracellular ATP. During the last hour of priming cells were treated with 1× caspase-1 FLICA reagent. Cells were washed and fixed then processed for immunofluorescence against (A) NLRP3 and (B) ASC. Three example cells are shown for each co-localization pair. Arrows indicate inflammasome foci. Scale bar represents 10 µm.

**Figure S2. KS6 sensor cellular response is insensitive to osmolarity effects.** LPS-primed J774A.1 macrophage cells were primed for 4 hours with 1 µg/mL E. coli O111:B4 LPS prior to staining with 5 µM KS6 potassium sensor. Cells were pre-treated for 15 minutes with 25 µM A438079, 130 mM KCl or 130 mM NaCl where indicated prior to stimulation. Cells were imaged by confocal microscopy and stimulated with 3 mM extracellular ATP at the denoted time. Traces represent the mean and standard error for 20 individual cells in each condition. Results are representative of at least 2 experiments.
